# The Century's Progress in Chemistry

**Published:** 1897-12

**Authors:** Henry Smith Williams


ARTICLE V.

### THE CENTURY'S PROGRESS IN CHEMISTRY.

BY HENRY SMITH WILLIAMS, M. D.

Small beginnings have great endings—sometimes. As a case in point, note what came of the small original effort of a self-trained back-country Quaker youth named John Dalton, who along toward the close of the last century became interested in the weather, and was led to construct and use a crude rain-gauge to test the amount of the waterfall. The simple experiments thus inaugurated led to no fewer than two hundred thousand recorded observations regarding the weather, which formed the basis for some of the most epochal discoveries in meteorology, as we have seen. But this was only a beginning. The simple rain-gauge pointed the way to the most important generalization of our century in a field of science with which, to the casual observer, it might seem to have no alliance whatever. The wonderful theory of atoms, on which the whole gigantic structure of modern chemistry is founded, was the logical outgrowth, in the mind of John Dalton, of those early studies in meteorology.

The way it happened was this: From studying the rain-fall, Dalton turned naturally to the complimentary process of evaporation. Hr was soon led to believe that vapor exists in the atmosphere as an independent gas. But since two bodies cannot supply the same space at the same time, this implies that the various atmospheric gases are really composed of discrete particles. These ultimate particles are so small that we cannot see them—cannot, indeed, more than vaguely imagine them—yet each particle of vapor, for example, is just as much a portion of water as if it were a drop out of the ocean, or, for that matter, the ocean itself. But again, water is a compound substance, for it may be separated, as Cavendish had shown, into the two elementary substances hydrogen and oxygen. Hence the atom of water must be composed of two lesser atoms joined together. Imagine an atom of hydrogen and one of oxygen. Unite them, and we have an atom of water; sever them, and the water no longer exists; but, whether united or separate, the atoms of hydrogen and of oxygen remain hydrogen and oxygen and nothing else. Differently mixed togethor or united, atoms produce different gross substances; but the elementary atoms never change their chemical nature—their distinct personality.

It was about the year 1803 that Dalton first gained a full grasp of the conception of the chemical atom. At once he saw that the hypothesis, if true, furnished a marvelous key to secrets of matter hitherto insoluble—questions relating to the relative proportions of the atoms themselves. It is known, for example, that a certain bulk of hydrogen gas unites with a certain bulk of oxygen gas to form water. If it be true that this combination consists essentially of the union of atoms one with another (each single atom of hydrogen united to a single atom of oxygen), then the relative weights of the original masses of hydrogen and of oxgen must be also the relative weights of each of their respective atoms. If one pound of hydrogen unites with five and one-half pounds of oxygen (as, according to Dalton's experiments, it did), then the weight of the oxygen atom must be five and one-half times that of the hydrogen atom. Other compounds may plainly be tested in the same way. Dalton made numerous tests before he published his theory. He found that hydrogen enters into compounds in smaller proportions than any other element known to him, and so, for convenience, determined to take the weight of the hydrogen atom as unity. The atomic weight of oxygen then becomes (as given in Dalton'r first table of 1803) 5.5; that of water (hydrogen plus oxygen) being of course 6.5. The atomic weights of about a score of substances are given in Dalton's first paper, which was read before the Literary and Philosophical Society of Manchester, October 21, 1803. I wonder if Dalton himself, great and acute intellect though he had, suspected, when he read that paper, that he was inaugurating one of the most fertile movements ever entered on in the whole history of science?

Be that as it may, it is certain enough that Dalton's contemporaries were at first little impressed with the novel atomic theory. Just at this time, as it chanced, a dispute was waging in the field of chemistry regarding a matter of empirical fact which must necessarily be settled before such a theory as that of Dalton could even hope for a hearing. This was the question whether or not chemical elements unite with one another always in definite proportions. Berthollet, the great co-worker with Lavoisier, and now the most authoritative of living chemists, contended that substances combine in almost indefinitely graded proportions between fixed extremes. He held that solution is really a form of chemical combination—a position which, if accepted, left no room for argument.

But this contention of the master was most actively in particular by Louis Joseph Provost, and all chemists of repute were obliged to take sides with one or the other, For a time the authority of Berthollet held out against the facts, but at last accumulated evidence told for Proust and his followers, and toward the close of the first decade of our century it came to be generally conceded that chemical elements combine with one another in fixed and definite proportions.

More than that. As the analysts were led to weigh carefully the quantities of combining elements, it was observed that the proportions are not onl definite but that they bear a very curious relation to one another, If element A combines with two different proportions of element B to form two compounds, it appeared that the weight of the larger quantity of B is an exact multiple of that of the smaller quantity. This curious relation was noticed by Dr. Wollaston, one of the most accurate of observers, and a little later it was confirmed by Johan Jakob Berzelius, the great Swedish Chemist, who was to be a dominating influence in the chemical world for a generation to come. But this combination of elements in numerical proportions was exactly what Dalton had noticed as early as 1802, and what had led him directly to the atomic weights. So the confirmation of this essential point by chemists of such authority gave the strongest confirmation to the atomic theory.

During these same years the rising authority of the French chemical world, Joseph Louis Gay-Lussac, was conducting experiments with gases, which he had undertaken at first in conjunction with Humboldt, but which later on were conducted independently. In 1809, the next year after the publication of the first volume of Dalton's "New System of Chemical Philosophy," Gay Lussac published the results of his observations, and among other things brought out the remarkable fact that gases, under the same conditions as to temperature and pressure, combine always in definite numerical proportions as to volume. Exactly two volumes of hydrogen, for example, combine with one volume of oxygen to form water. Moreover, the resulting compound gas always bears a simple relation to the combining volumes. In the case just cited the union of two volumes of hydrogen and one of oxygen results in precisely two volumes of water vapor.

Naturally enough the champions of the atomic theory seized upon these observations of Gay-Lussac as lending strong support to their hypothesis—all of them, that is, but the curiously self-reliant and self-sufficient author of the atomic theory himself, who declined to accept the observations of the French chemist as valid. Yet the observations of Gay-Lussac were correct, as countless chemists since then have demonstrated anew, and his theory of combination by volumes became one of the foundation-stones of the atomic theory, despite the opposition of the author of that theory.

The true explanation of Gay-Lussac's law of combination by volumes was thought out almost immediately by an Italian savant, Amadeo Avogadro, and expressed in terms of the atomic theory. The fact must be, said Avogadro, that under similar physical conditions every form of gas contains exactly the same number of ultimate particles in a given volume. Each of these ultimate physical particles may be composed of two or more atoms (as in the case of water vapor), but such a compound atom conducts itself as if it were a simple and indivisible atom, as regards the amount of space that separates it from its fellows under given conditions of pressure and temperature. The compound atom, composed of two or more elementary atoms, Avogadro proposed to distinguish, for purposes of convenience, by the name molecule. It is to the molecule, considered as the unit of physical structure, that Avogadro's law applies.

This vastly important distinction between atoms and molecules, implied in the law just expressed, was published in 1811. Four years later, the famous French physicist Ampere outlined a similar theory, and utilized the law in his mhthematical calculations. And with that the law of Avogadro dropped out of sight for a full generation. Little suspecting that it was the very key to the inner mysteries of the atoms for which they were seeking, the chemists of the time cast it aside, and let it fade from the memory of their science.

This, however, was not strange, for of course the law of Avogadro is based on the atomic theory, and in 1811 the atomic theory was itself still being weighed in the balance. The law of multiple proportions found general acceptance as an empirical fact; but many of the leading lights of chemistry still looked askance at Dalton's explanation of this law. Thus Wollaston, though from the first he inclined to acceptance of the Daltonian view, cautiously suggested that it would be well to use the non-committal word "equivalent" instead of "atom;" and Davy, for a similar reason, in his book of 1812, speaks only of "proportions," binding himself to no theory as to what might be the nature of these proportions.

At least two great chemists of the time, however, adopted the atomic view with less reservation. One of these was Thomas Thomson, professor at Edinburgh, who in 1807 had given an outline of Dalton's theory in a widely circulated book, which first brought the theory to the general attention of the chemical world. The other, and even more noted advocate of the atomic theory, Johan Jakob Berzelius. This great Swedish chemist at once set to work to put the atomic theory to such tests as might be applied in the laboratory. He was an analyst of the utmost skill, and for years he devoted himself to the determination of the combining weights, "equivalents," or "proportions" of the different elements. These determinations, in so far as they were accurately made, were simple expressions of empirical facts, independent of any theory; but gradually it became more and more plain that these facts all harmonize with the atomic theory of Dalton. So by common consent the proportionate combing weights of the elements came to be known as atomic weights—the name Dalton had given them from the first—and the tangible conception of the chemical atom as a body of definite constitution and weight gained steadily in favor.

From the outset the idea had the utmost tangibility in the mind of Dalton. He had all along represented the different atoms by geometrical symbols—as a circle for oxygen, a circle enclosing a dot for hydrogen, and the like—and had represented compounds by placing these symbols of the elements in juxtaposition. Berzelius proposed to improve upon this method by substituting for the geometrical symbol the initial of the Latin name of the element represented—O for oxygen, H for hydrogen, and so on—a numerical coefficient to follow the letter as an indication of the number of atoms present in any given compound. This simple system soon gained general acceptance, and with slight modifications it is still universally employed. Every school boy now is aware that $H_2O$ is the chemical way of expressing the union of two atoms of hydrogen with one of oxygen to form a molecule of water. But such a formula would have had no meaning for the wisest chemist before the day of Berzelius.

The universal fame of the great Swedish authority served to give general currency to his symbols and atomic weights, and the new point of view thus developed led presently to two important discoveries, which removed the last lingering doubts as to the validity of the atomic theory. In 1819 two French physicists, Dulong and Petit, while experimenting with heat, discovered that the specific heats of solids (that is to say, the amount of heat required to raise the temperature of a given mass to a given degree) vary inversely as their atomic weights. In the same year Eilhard Mitscherlich, a German investigator, observed that compounds having the same number of atoms to the molecule are disposed to form the same angles of crystallization—a property which he called isomorphism.

Here, then, were two utterly novel and independent sets of empirical facts which harmonize strangely with the supposition that substances are composed of chemical atoms of a determinate weight. This surely could not be coincidence—it tells of law. And so as soon as the claims of Dulong and Petit and of Mitscherlich had been substantiated by other observers, the laws of the specific heat of atoms, and of isomorphism, took their place as new levers of chemical science. With the aid of these new tools an impregnable breastwork of facts was soon piled about the atomic theory. And John Dalton, the author of that theory, plain provincial Quaker, working on to the end in semi retirement, because known to all the world and for all time as a master of masters.—Extract from "Harper's Magazine, October," 1897, *Western Dental Journal.*

